# Clinical response to pandemic h1n1 influenza virus from a fatal and mild case in ferrets

**DOI:** 10.1186/s12985-015-0272-x

**Published:** 2015-03-26

**Authors:** Pamela Martínez-Orellana, Jaume Martorell, Beatriz Vidaña, Natalia Majó, Jorge Martínez, Ana Falcón, Ariel Rodríguez-Frandsen, Inmaculada Casas, Francisco Pozo, Lourdes García-Migura, Blanca García-Barreno, Jose A Melero, Lorenzo Fraile, Amelia Nieto, Maria Montoya

**Affiliations:** Centre de Recerca en Sanitat Animal (CReSA), UAB-IRTA, Campus de la Universitat Autònoma de Barcelona, Bellaterra, Barcelona Spain; Departament de Medicina i Cirurgia Animals, Universitat Autònoma de Barcelona (UAB), Barcelona, Spain; Departament de Sanitat i Anatomia Animals, Universitat Autònoma de Barcelona (UAB), Barcelona, Spain; Centro Nacional de Biotecnología, CSIC. Campus de la Universidad Autónoma, Cantoblanco, Madrid Spain; CIBER de Enfermedades Respiratorias, Mallorca, Illes Baleares Spain; Centro Nacional de Microbiología, ISCIII, Majadahonda, Madrid Spain; Universitat de Lleida, Lleida, Spain; Institut de Recerca i Tecnologia Agroalimentàries (IRTA), Barcelona, Spain; Present address: The Pirbright Institute, Ash Road, Woking, GU24 0NF, Pirbright, UK; Present address: Infectious and Inflammatory Disease Center, Sanford-Burnham Medical Research Institute, La Jolla, CA USA

**Keywords:** Pandemic A(H1N1)pdm09, Clinical response, Ferrets

## Abstract

**Background:**

The majority of pandemic 2009 H1N1 (A(H1N1)pdm09) influenza virus (IV) caused mild symptoms in most infected patients, however, a greater rate of severe disease was observed in healthy young adults and children without co-morbid conditions. The purpose of this work was to study in ferrets the dynamics of infection of two contemporary strains of human A(H1N1)pdm09 IV, one isolated from a patient showing mild disease and the other one from a fatal case.

**Methods:**

Viral strains isolated from a patient showing mild disease-M (A/CastillaLaMancha/RR5661/2009) or from a fatal case-F (A/CastillaLaMancha/RR5911/2009), both without known comorbid conditions, were inoculated in two groups of ferrets and clinical and pathological conditions were analysed.

**Results:**

Mild to severe clinical symptoms were observed in animals from both groups. A clinical score distribution was applied in which ferrets with mild clinical signs were distributed on a non-severe group (NS) and ferrets with severe clinical signs on a severe group (S), regardless of the virus used in the infection. Animals on S showed a significant decrease in body weight compared to animals on NS at 4 to 7 days post-infection (dpi). Clinical progress correlated with histopathological findings. Concentrations of haptoglobin (Hp) and serum amyloid A (SAA) increased on both groups after 2 dpi. Clinically severe infected ferrets showed a stronger antibody response and higher viral titres after infection (p = 0.001).

**Conclusions:**

The severity in the progress of infection was independent from the virus used for infection suggesting that the host immune response was determinant in the outcome of the infection. The diversity observed in ferrets mimicked the variability found in the human population.

## Introduction

Influenza A viruses are classified as members of the family *Orthomyxoviridae*. They are classified according their haemagglutinin (HA) and neuraminidase (NA) molecules, which are the basis of the antigenicity [1]. They can produce significant respiratory disease in humans and in a whole range of avian and mammals species. In some occasions, simultaneous infection in a susceptible species by more than one influenza A viruses can lead to gene mixing, or reassortment. These processes can originate a novel influenza virus strain, against which human population would have few or no existing immunity [2]. A new influenza A virus from H1N1 subtype, possessing high transmissibility but relatively low virulence, emerged in 2009 (A(H1N1)pdm09) rapidly spreading across the entire globe and causing the first pandemic of the 21^st^ century. It carried genes of avian, swine and human IV [3,4]. Nowadays, A(H1N1)pdm09 still remain being an important issue of public health, and in general terms, A(H1N1)pdm09 infection can cause mild and self-limiting symptoms [5]. However, in some cases the disease progresses towards severity, exhibiting symptoms that have been characterized by severe or complicated illness, often requiring hospitalization and mortality [6-8].

Previous studies in our group indicated that the virus isolated from the fatal case replicates faster, induces higher levels of cytokines in human alveolar lung epithelial cells and is more pathogenic in a murine model *in vivo*, compared with the virus obtained from the patient with mild disease [9]. Even though mice have been widely used as animal model for influenza studies, it is not a natural host for influenza virus infection. On the other hand, ferrets *(Mustela putorius furo),* are well recognized as an animal model for IV. Together with their similarities with humans in the respiratory tract, lung physiology and airway morphology, ferrets also develop clinical signs to influenza infections similar to humans, usually restricted to the upper respiratory tract including sneezing, nasal discharge, malaise, and pyrexia. In general terms, ferrets develop mild clinical signs, and occasionally pneumonia, which is more severe or even fatal depending on the age of the host and/or the strain of the virus [10-12]. On the other hand, as an outbreed animal, the response developed in ferrets when infected with IV goes from mild clinical signs to severe or even a fatal result, similar to the response found in patients.

This study was focused on the clinical response exhibited on infected ferrets with the two viruses which showed differential virulence in mice. Thus, a thorough clinical scoring was established, enlarging the spectrum of clinical signs usually analyzed [13]. Also, acute phase proteins such as haptoglobin and serum amiloid albumin were studied for the first time in IV infected ferrets. Our results demonstrate that, contrary to the outcome in mice, the severity in the progress of infection was independent from the virus used for infection, indicating that the host response was determinant in the outcome of the infection.

## Materials and methods

### Ethics statement

All experiments were performed under a reviewed and approved protocol (n° 1976) by “Comissió d’Ètica en l’Experimentació Animal I Humana de la Universitat Autònoma de Barcelona”. Ferrets were housed in groups on experimental isolation rooms at the biosafety level 3 facilities of the Centre de Recerca en Sanitat Animal (CReSA, Barcelona, Spain).

### Virus and cells

Two distinct IV named A/CastillaLaMancha/RR5661/2009 (M) and A/CastillaLaMancha/RR5911/2009 (F), were isolated at the National Influenza Centre (CNM, ISCIII) from respiratory samples sent by the Spanish Influenza Surveillance System for virological characterization. Virus M was isolated from a 23 years old male patient who showed mild clinical signs of influenza, and virus F was isolated from a 35 years old female patient who developed an infection with fatal consequence. Neither of the patients presented previous pathology at the moment of infection. Both viruses were thoroughly described in Rodriguez et al. manuscript [9].

Madin-Darby Canine kidney (MDCK) cells were cultured in Dulbecco's Modified Eagle Medium (DMEM) supplemented with 5% fetal bovine serum (FBS), 100UI/ml penicillin and 100ug/ml streptomycin, 2 mM glutamine.

IV M and F were grown in MDCK three times. M virus had a titre of 10^8,3^ TCID50/ml and F virus had a titre of 10^8,2^ TCID50/ml.

### Animals and infection

Fourteen adult ferrets (*Mustela putorius furo*) under 24 month-old were numbered from 1 to 14, and were randomly selected from a stable, purposely bred colony (Isoquimen, Spain)*.* Ferrets were randomly assigned to different experimental groups. The groups were separated into experimental isolation rooms, and kept for one week in acclimation. Ferrets were kept in standard housing cages and were provided with commercial food pellets and tap water *ad libitum* throughout the experiment. Animals were divided into three groups. The control group (1) included two ferrets, number 1(male) and number 2 (female) and they were inoculated intratracheally with PBS. Group 2 included animals infected with M strain numbered from 3 to 8 and was integrated by 4 males (ferrets 3 to 6) and two females (ferrets 7 and 8). Group 3 included animals infected with F strain numbered from 9 to 14 and they were all males. Ferrets were intratracheally inoculated with 200 μl containing 10^6^ TCID_50_ of the corresponding virus. All ferrets were proven seronegative at 0 dpi by ID ScreenH Influenza A Antibody Competition ELISA (ID VET, France).

### Clinical score

Ferrets were monitored daily for clinical signs and changes in temperature and body weight. Clinical signs were recorded according to parameters defined in Table 1.

**Table 1:** **Clinical Score**

**Clinical score**	**0**	**1**	**2**	**3**
**Playful**	normal	mild apathy	apathetic	
**Arched back**	normal	arched		
**Bristly hair**	no bristly	bristly tail	bristly tail and back	all body bristly
**Sneezing**	no	sporadic	frecuent	
**Nasal secretion**	no secretion	low	abundant	
**Conjunctival secretion**	no secretion	low	abundant	
**Breathing rate**	normal (40–50)	50-70	70<	
**Auscultation**	normal	anomalous		
**Lost weight**	no lost weight	(1-5%)	(5-10%)	(>10)
**Temperature**	normal (37.5-39,4)	hyperthermia (39.5-40°C)	(>40)	<37
**Mucous**	normal	congestive		
**Heart rate**	<250	250-300	300 <	

Animals were monitored daily at the same time for clinical observations.

Changes in rectal temperature and body weight were measured at approximately the same time each day. Any animal losing 25% of its day 0 body weight, exhibiting a scoring of more than 20 points or determined to be in a moribund state was humanely euthanized.

### Sampling

Samples of blood and serum were collected at 0, 2, 4, 7, 10 and 14 dpi. Two animals of groups 2 and 3 were euthanized at 4, 7 and 14 dpi. From these animals samples of lung, trachea and bronchoalveolar lavage (BAL) were collected.

All the ferrets were sedated with 0.5 mg/kg butorphanol administered subcutaneously for blood collection. 2 ml of blood were taken from Cava Cranial Vein at 0, 2, 4, 7, 10 and 14 dpi. All samples were collected into a 1 ml blood-heparine tubes for Acute Phase Protein (APP) determination. Heparin blood samples were centrifugated at 3000 rpm for 10 minutes at 4°C to separate plasma sample. Plasma samples were store in −20°C.

### Acute phase proteins

Acute phase proteins were determined by commercial ELISAs according to the manufacturer’s recommendation (Haptoglobin Assay and Multispecies Serum Amyloid A Immunoassay, both from Tridelta Development Ltd, County Kildare, Ireland). Serum samples were tested in duplicate.

### Hemagglutination Inhibition (HI) Assay

Antibodies against IV were measured using a HI assay using chicken red blood cells (RBC) and 4 hemagglutination units of either M and F viruses. Before assay, sera were treated overnight at 37°C with four volumes of Receptor Destroying Enzyme (RDE) (Sigma-Aldrich SA, Madrid, Spain) solution (100 U/ml) to remove non-specific inhibitors of hemagglutination. The following day, serum samples were incubated for 30 min at 56°C after the addition of five volumes 1.5% sodium citrate. Finally, one volume of a 50% suspension of RBC was added and incubated for 1 hour at 4°C. Known positive and negative sera were used as controls. HI titres of >40 were considered positive.

### Histopathology and Immunohistochemistry

A complete necropsy was performed on all animals immediately after euthanasia. No major lesions were observed macroscopically in any organ at necropsy. Lung samples were taken in a standardized way, not guided by changes as seen in the gross pathology. Tissue samples were fixed for 48 hours in neutral-buffered 10% formalin. They were then embedded in paraffin wax, sectioned at 3 μm, and stained with haematoxylin and eosin (HE) for histopathological assessment.

Influenza A virus antigen detection was performed in tissues stained with a primary antibody against the influenza A nucleoprotein (NP). Briefly, paraffin-embedded samples were sectioned at 3 μm thick, dewaxed and treated with 3% H_2_O_2_ in methanol to eliminate the endogenous peroxidase. Then, sections were treated with protease at 37°C for 10 minutes and blocked with 2% bovine serum albumin (85040C, Sigma-Aldrich Quimica, S.A., Spain) for one hour. Later, tissues were incubated with the primary monoclonal antibody anti-NP Influenza A virus (ATCC, HB-65, H16L-10-4R5) diluted 1:250, at 4°C overnight. After being rinsed, the samples were incubated with biotinylated goat anti-mouse IgG secondary antibody (Dako, immunoglobulins AS, Glostrup, Denmark), followed by incubation with avidin-biotin-peroxidase complex (ABC) (Thermo Fisher Scientific, Rockford, IL, USA). The reaction was developed with 3,3'-Diaminobenzidine tetrahydrochloride (DAB) (Sigma-Aldrich, Madrid, Spain) at room temperature, followed by counterstaining with Mayer's haematoxylin. Swine lung sections from pig experimentally infected with Influenza A virus, were used as positive controls. Same sections in which the specific primary antibodies were substituted with PBS were used as negative controls.

### Viral load

Tissue samples of ferrets were collected and snap frozen on dry ice and stored at −80°C until further processing. Tissue samples were weighed and subsequently homogenized and centrifuged briefly. Infectivity of M and F viruses was determined by plaque assay in MDCK cells. MDCK cells plated in 12-well tissue cultures plates were inoculated with 0.1 ml of 10-fold dilutions for each homogenized tissues (10^1^–10^6^ PFU) diluted in Phosphate Buffer Saline (PBS) (AttendBio Research, S.L, Barcelona, Spain) and 1% bovine serum albumin (BSA) (Sigma-Aldrich SA, Madrid, Spain) at room temperature. Samples were adsorbed to MDCK cells for 1 hour, with shaking every 15 minutes. After then, inoculums were aspirated and the cells were washed once with PBS (Lonza, Walkesville, USA). Wells were overlaid with 1.4% Noble agar (Becton Dickinson, France) mixed 1:1 with Minimum Essential Medium Eagle (MEM) (Sigma-Aldrich SA, Madrid, Spain) supplemented with 100UI/ml penicillin and 100ug/ml streptomycin (Invitrogen ®, Barcelona, Spain) and 0.5 μg/ml of bovine trypsin (Sigma-Aldrich SA, Madrid, Spain). Plates were inverted and incubated for 4 days. Cells were fixed for 20 min using 10% formalin (Sigma-Aldrich SA, Madrid, Spain) and then overlaid with 1% crystal violet (Anorsa, Barcelona, Spain). Cells were then washed with water to visualized plaques. Plaques were counted and compared to uninfected cells.

### Statistical analysis

All statistical analysis was performed using SPSS 15.0 software (SPSS Inc., Chicago, IL, USA). For all analyses, ferret was used as the experimental unit. The significance level (α) was set at 0.05 with statistical tendencies reported when *P* < 0.10. The Shapiro Wilk’s and the Levene test were used to evaluate the normality of the distribution of the examined quantitative variables, and the homogeneity of variances, respectively. It was not detected any continuous variable that had a normal distribution. Thus, a non-parametric test (Wilcoxon test) using the U Mann–Whitney test to compare each pair of values was used to compare the different values obtained for all the parameters (clinical, score, acute phase proteins, antibody response and viral load), between groups (NS and S) at all sampling times.

## Results

### Clinical score

During the course of the experiment, clinical observations scores were recorded daily according to the score set in Table 1. Infected ferrets started to show clinical signs such as decrease in activity level, nasal discharge and/or sneezing at 2 dpi which extended over the following 10 days in both infected groups. As an outbreed animals, ferrets from both infected groups showed high variability in the progress of the clinical infection. In order to analyse each individual ferret, animals were classified according to each clinical score: uninfected control (C) ferrets obtained less than 4 points, ferrets scoring within 6 to 11 were classified as non severe (NS) and animals scoring within 12 to 19 were classified as severe (S). Regardless of the virus used as inoculum, animal distribution was established as follow: control group (C) included two ferrets, non-severe group (NS) included seven ferrets, numbers 3, 10, 11, 12, 13, 14 (males) and 8 (female) and severe group (S) included five ferrets, numbers 4, 5, 6, 9 (males) and 7 (female) (Table 2). The proportion of severe and non severe affected animal did not correlate with the virus used for infection. Animals belonging to the S group paradoxically included most of the M infected animals: 4 animals were infected with M-virus whereas 1 animal was infected with F-virus. NS group included 5 animals infected with F-virus and 2 animals infected with M-virus.

**Table 2:** **Clinical score classification per animal**

**Inoculum**	**Animal number**	**Clinical score day 4 post-infection**	**Group**
MOCK	1	4	CONTROL
	2	4	CONTROL
A/CastillaLaMancha/RR5661/2009(M)	3	6	NON SEVERE
	4	19	SEVERE
	5	13	SEVERE
	6	17	SEVERE
	7	13	SEVERE
	8	11	NON SEVERE
A/CastillaLaMancha/RR5911/2009(F)	9	14	SEVERE
	10	11	NON SEVERE
	11	11	NON SEVERE
	12	10	NON SEVERE
	13	6	NON SEVERE
	14	11	NON SEVERE

Animals were classified according to each individual clinical score. Ferrets with less than 4 points remained as control (C), ferrets scoring within 6 to 11 were classified as non severe (NS) and animals scoring within 12 to 19 were classified as severe (S).

**Figure 1:**
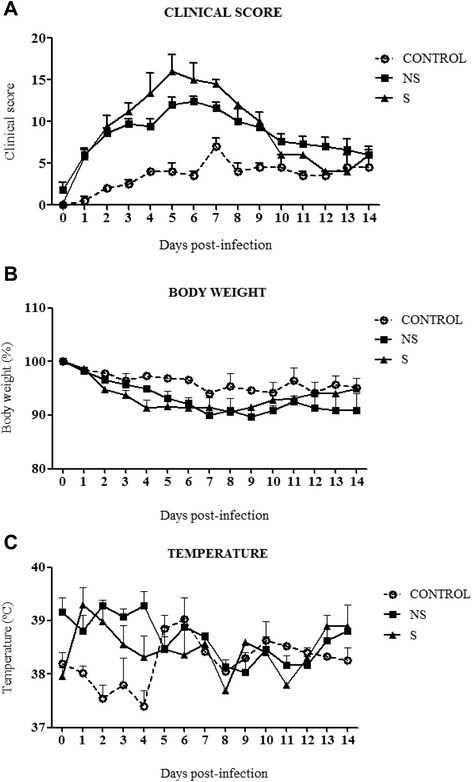
**Clinical signs of disease in ferrets following infection with M or F viruses.** Animals were monitored daily for clinical observations using a specific scoring system from Table 1. **(A)** Clinical score changes exhibited by S and NS group showing a statistically significant severe clinical score when compared with C animals at day 2, 3 and 4 post-infection (p < 0.05) **(B)** Percentage of weight loss on S and NS ferrets showing a significant decrease when compared with C group at day 4 post-infection. **(C)** Body temperature from animals belonging to C, NS and S. This parameter was not affected during infection experiment with any virus.

At 4 dpi, one ferret infected with M virus and one ferret infected with F virus lost around 25% of body weight and scored more than 20 points. Both animals were humanely euthanized. Clinical conditions were significantly affected on infected-ferrets throughout infection. Animals belonging to NS showed a statistically significant clinical score when compared with control animals at 2, 3 and 4 dpi (p < 0.05) (Figure 1A). A statistical tendency (p < 0.10) in higher clinical score was observed in S animals when compared with control group at 2, 3 and 4 dpi. On the following days: 5, 6 and 7 dpi, a tendency persisted within animals from NS when compared with control ferrets (Figure 1A). The group with severe clinical signs had a significant decrease in body weight (p < 0.05) when compared to NS at 2 dpi. At 4 dpi, there was a tendency (p < 0.10) reflected in higher percentage of weight loss on S when compared with NS. Ferrets with mild clinical score showed a tendency of decreased on body weight (p < 0.10) when compared with control group at 4 dpi (Figure 1B). Body temperature was not affected by infection with any virus, although some increase of temperature was observed in ferrets belonging to the S group at day 1 pi which returned to control levels by day 5 pi. Any change in temperature was statistically significant (Figure 1C).

### Histopathology and Immunohistochemistry

**Figure 2:**
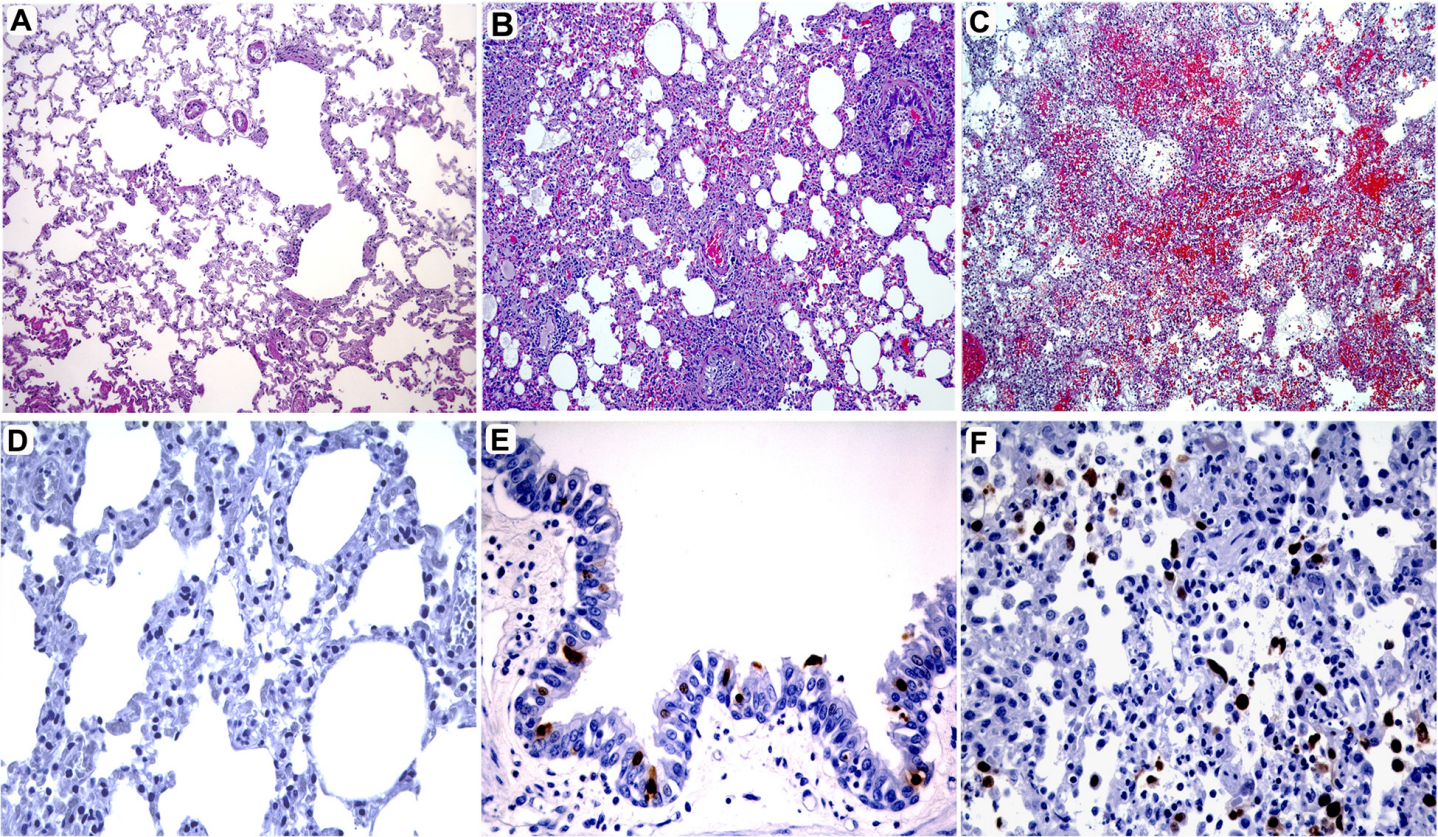
**Lung histopathology and NP inmunohistochemestry of ferrets with different clinical symptoms. (A)** Histopathology of a representative control ferret. Hematoxilin/Eosin (HE) stain (20x objective field); **(B)** Histopathology of a representative ferret which presented NS signs and bronchopneumonia. Bronchiolar-alveolar junction showing mild lymphoplasmacytic infiltration and bronchial epithelium slough. HE stain (20x objective field; **(C)** Histopathology of a representative ferret presenting S signs exhibiting severe clinical signs and interstitial pneumonia. Detail of the alveolar interstitium showing diffuse alveolar damage with presence of hyaline membranes, edema, large macrophagic infiltrate and hemorrhage. HE stain (20x objective field); **(D)** NP staining in a representative control ferret. Haematoxylin counterstain (40x objective field); **(E)** NP staining in a representative ferret belonging to the NS group showing viral antigen present only in bronchial epithelia. Haematoxylin counterstain (40x objective field); **(F)** NP staining in a representative ferret belonging to the S group, showing viral antigen staining in pneumocytes and alveolar macrophages. Haematoxylin counterstain (40x objective field).

Most significant lesions were observed in lungs from infected animals. Interstitial pneumonia, also called diffuse alveolar damage (Figure 2C), characterized by an acute inflammation with large amounts of neutrophils, macrophages and hyaline membranes filling the alveolar lumen was observed in lungs of ferrets presenting severe clinical symptoms (S). Mild bronchopneumonia (Figure 2B) or bronchitis, consistent with a mild exudative lesion with suppurative or lymphoplasmacytic infiltration and bronchial epithelial necrosis was observed in animals which reported only mild clinical signs (NS). No histological abnormalities were found in control ferrets (Figure 2A).

Presence of NP viral antigen detected by immunohistochemistry (IHC) was only observed in animals sacrificed at 4 dpi (Figure 2D-F). Presence of viral antigen correlated with the grade of pathological lesion observed. Animals which presented more severe pathological lesions showed higher amounts of viral antigen by IHC when compared the lesions and NP staining from animals belonging to the NS group with their counterparts from the S group. In the S group animals, viral antigen was mainly observed in the bronchiolar epithelium, in the surface and glandular epithelium in the bronchi and interstitially in the alveolar septa and alveoli, mainly located in the alveolar epithelium. Animals which presented mild pathological lesions showed a lesser amount of NP positive cells or did not show any positive cells at all; in these animals positivity was restricted to bronchiolar epithelial cells. In summary, animals presenting more severe pathological lesions showed higher amounts of viral antigen mainly observed in the bronchiolar and bronchial epithelium, glandular epithelium in the bronchi and interstitially in the alveolar septa and alveoli, mainly located in the alveolar epithelium.

### Acute phase proteins (APP)

**Figure 3:**
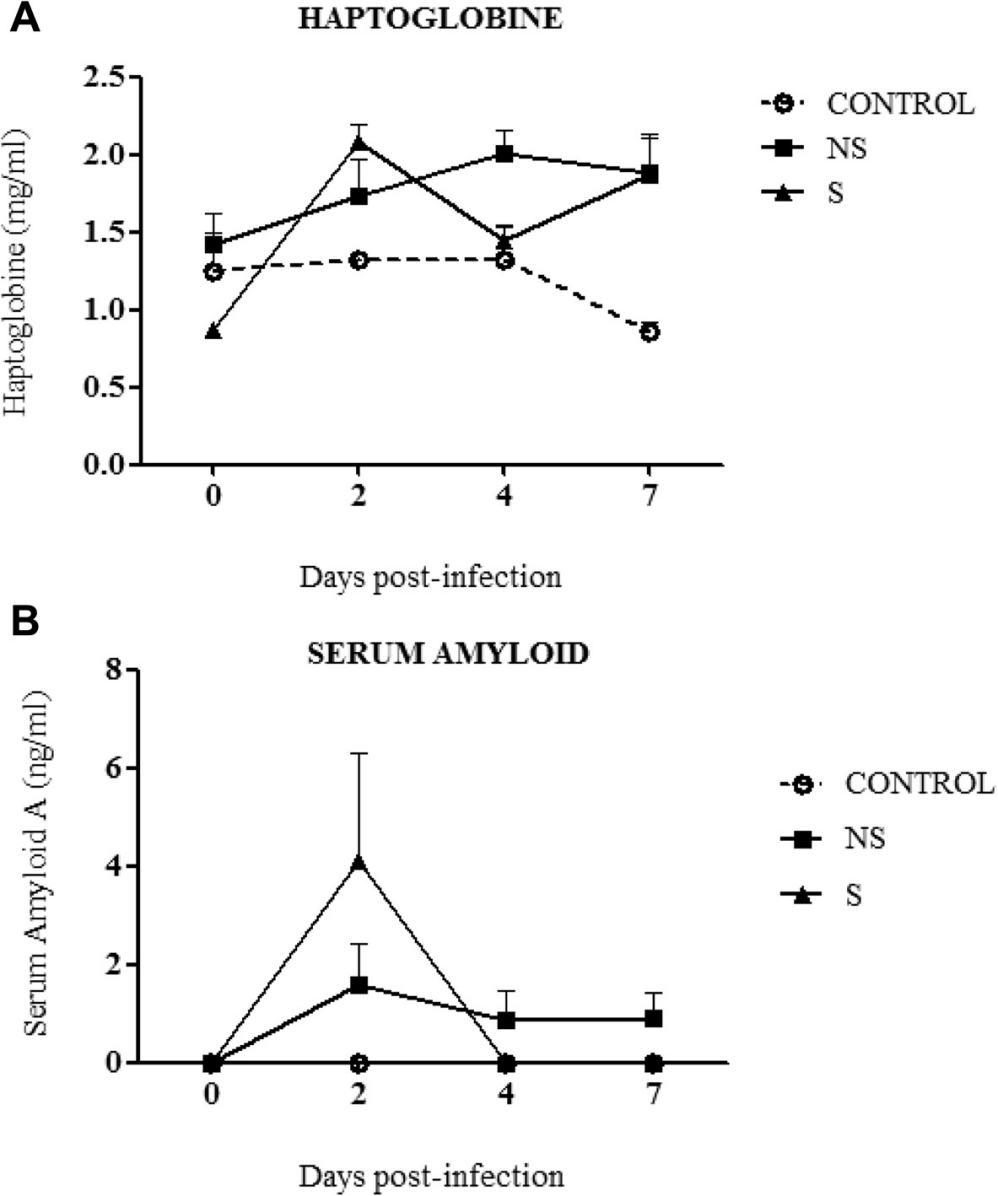
**Concentrations of acute phase proteins in serum of influenza virus infected ferrets (A) Haptoblobine (Hp) and (B) Serum Amyloid (SAA) of two groups of ferrets presenting S and NS symptoms of the disease, before and at various time points after intratracheal infection with A(H1N1)pdm09 viruses.**

Haptoglobin (Hp) being an APP, may increase in plasma in any inflammatory process like IV infection. Indeed, an increase in Hp levels was reported during influenza in pigs caused by A(H1N1)pdm09 virus [14,15]. In ferrets, pre-inoculation individual levels of Hp were found to be below 1.94 mg/ml. The highest individual level after infection reached 2.44 mg/ml (at 2 dpi) in an animal presenting mild symptoms of the disease. No significant changes in the concentration of Hp were observed during the study period. However, the mean concentrations of Hp increased from 0.87 to 2.08 (at 2 dpi) and from 1.42 to 2.01 (at 4 dpi) in the S and NS, respectively (Figure 3A).

Serum amyloid A (SAA) is another important APP that has also been reported to increase in serum from humans and horses after influenza infection [14,15]. In ferrets, an increase of SAA after infection with the virus was detected when compared to their control counterparts only at 2 dpi. Large variations in SSA concentrations were observed between animals presenting severe symptoms of the infection at 2 dpi. At this time point, the highest mean peak level reached 4.1 ng/ml. However, the SAA concentration had decreased to zero in the group presenting severe symptoms at day 4 dpi, in the mild group the concentration had stagnated (Figure 3B).

### Antibody response

Sera from 0, 10 and 14 dpi were examined for the presence of specific antibodies against influenza NP. All infected animals showed a positive response at 10 and 14 dpi (Table 3). Antibody response to M and F viruses was determined by hemagglutination inhibition (HAI) assay in sera from 2, 4, 7, 10 and 14 dpi. Control ferrets shown to be seronegative at all time-points. Animals belonging to NS and S revealed an early presence of hemagglutinin inhibiting antibodies for M and F viruses as soon as 7 dpi. There was a strong HI antibody response to both viruses in sera of infected-groups at 10 and 14 dpi (Table 3). There was not significant differences within infected-groups. Control group showed statistically significant differences with infected groups at all time points.

**Table 3:** **Antibody response against F and M was determined by NP ELISA and HIAAt days 10 and 14 serum samples were collected to determine positive Influenza A samples by NP ELISA**

**Group**	**Animal number**	**Virus**	**ELISA NP**		**IHA**					**IHA**				
					**A/CastillaLaMancha/RR5911/2009 (F)**					**A/CastillaLaMancha/RR5661/2009 (M)**				
			10dpi	14dpi	2dpi	4dpi	7dpi	10dpi	14dpi	2dpi	2dpi 4dpi	7dpi	10dpi	14dpi
C	1	mock	-	-	<40	<40	<40	<40	<40	<40	<40	<40	<40	<40
	2	mock	-	-	<40	<40	<40	<40	<40	<40	<40	<40	<40	<40
NS	3	M	+	+	<160	<160	<10.240	<5.120	<10.240	<80	<40	<5.120	<10.240	<20.480
	10	F	+	+	<320	<320	<10.240	<10.240	<10.240	<80	<160	<10.240	<20.480	<20.480
	11	F	+	+	<320	<320	<5.120	<10.240	<5.120	<160	<160	<5.120	<20.480	<5.120
S	4	M	†	†	<160	<320	†			<80	<160	†		
	5	M	+	+	<320	<320	<10.240	<10.240	<10.240	<80	<160	<10.240	<10.240	<10.280
	9	F	†	†	<320	†				<80	†			

Antibody response against F and M virus was determined by HIA analyzing serum samples from 2, 4, 7, 10 and 14 dpi. † Animals were sacrificed following the protocol described in material and methods.

### Viral load

**Figure 4:**
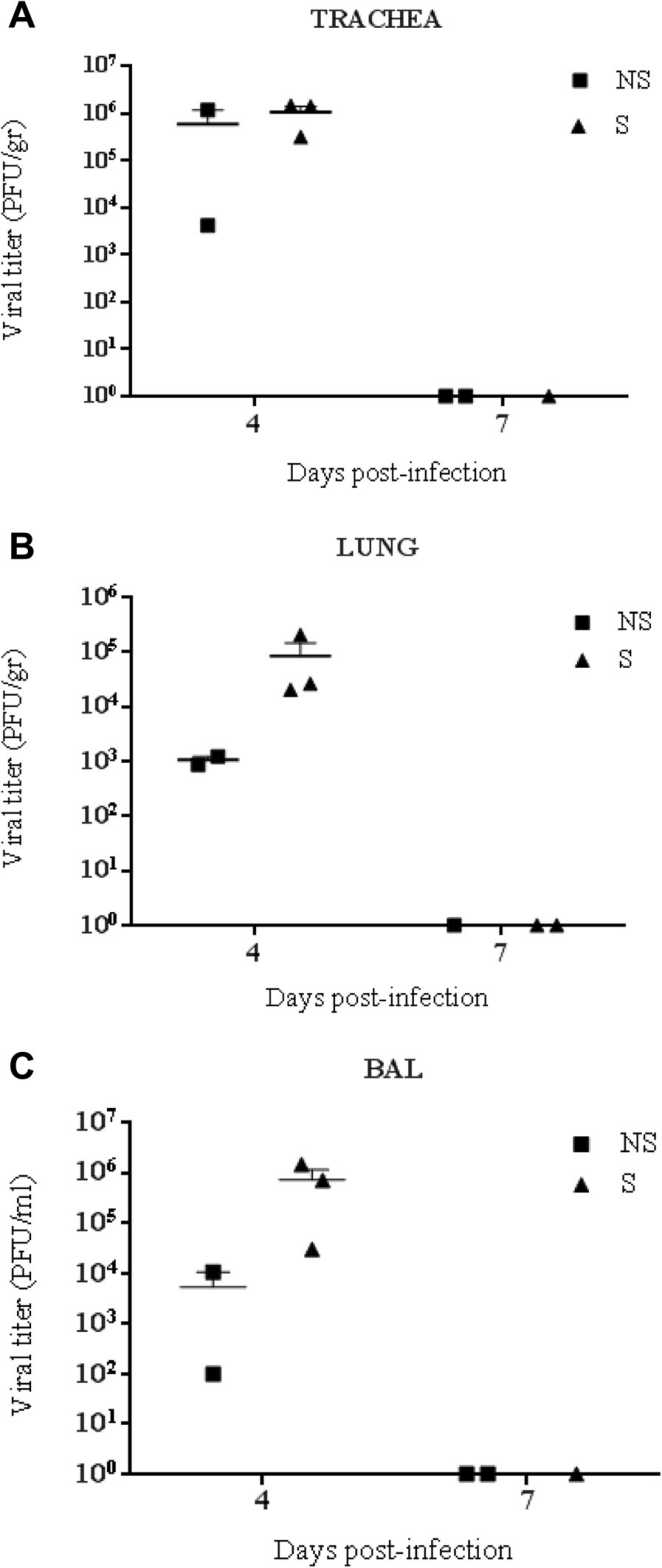
**Viral replication in trachea, lungs and broncheo alveolar lavage (BAL) of infected ferrets.** At 4 and 7 dpi, samples of trachea, lung and BAL were collected to measure viral titers. **(A)** Virus titers in trachea; **(B)** Lung titers, **(C)** Viral titers in BAL.

To test the presence of infectious virus particles, samples of trachea, lung and broncheo alveolar lavage (BAL) were collected at 4 and 7 dpi. Control animals were negative. Data of viral titres of trachea, lung and BAL was analyzed as a set including the three different tissues (Figure 4). Viral titres were similar at 4 dpi in trachea when values from animals in S group and NS group were compared. On the contrary, ferrets with severe clinical signs at 4 dpi exhibited higher viral titres than their counterparts in the NS group in the lungs and BAL. This difference was statistically significant (p = 0.01). No virus was detected in trachea, lung or BAL at 7dpi.

## Discussion

On April of 2009, a new type of influenza H1N1 came to the world's attention due to their characteristics of rapid spread among humans all over the world. Patients affected by A(H1N1)pdm09 infection developed a whole range of clinical features. Most patients infected by the virus experienced uncomplicated illness, however, a small subset of patients developed a severe disease which may be related to their individual susceptibilities. Influenza pandemic viruses used for the infection in our study were collected from two young adults patients with unknown previous comorbid conditions as asthma, pregnancy, diabetes or obesity, which were reported to be highly associated with the development of critical illness in H1N1pdm infected patients [16-19]. Also, other investigations attempt to study specific markers associated to severity during H1N1pdm infection in order to described unknown underlying immunosupressory conditions [20]. Ferrets were selected to mimic the variability within a heterogeneous population. Our study underlined that ferrets are an appropriate animal model to study the individual clinical characteristics observed on humans infected with the A(H1N1)pdm09 virus. Ferrets were infected with two strains of A(H1N1)pdm09 2009 virus, one isolated from a patient exhibiting mild clinical signs of infection, and the other one from a patient with a fatal outcome. Genetic characterization of both virus was already discussed elsewhere [9]. There were eleven males and three females with ages ranging from one to two years of adult life. Surprisingly, during the course of the experiment, ferrets from both infected groups, showed a clinical score severity that was independent from the virus used for infection. Animals infected with M virus presented mild to severe clinical signs and one ferret presented a critical condition and was euthanized for humanitarian reasons. The same picture was observed in the F-infected group. Control ferrets scored up to 4 points that were attributed to the mock treatment. These findings differ from the results obtained in a previous work in which the same two IV strains were used to infect mice. The data in the murine model indicated that F virus was more pathogenic, shown by the morbidity and mortality rates observed in the F-infected mice [9]. However, in the present work, due to the clinical observations registered during the ferret infection, the data were analysed dividing all animals by the clinical score. In such manner, each animal was treated by their individual clinical condition observed during infection. Changes on weight, body temperature and activity are the most common parameters observed following infection used to evaluate the clinical profiles associated with influenza infection in ferrets. For example, the activity scoring, generally used to assess activity changes in infected ferrets are based on the scoring system described by Reuman et al. [21] and Zitzow et al. [22]. In order to perform a more comprehensive evaluation, extensive data were recorded including a whole range of clinical signs (Table 1). The results in this work showed that the clinically severe animals showed a pattern of clinical signs similar to that commonly founded on ferrets infected with a HPAI, whereas the animals presenting mild clinical signs response exhibited similar symptoms to those infected with a seasonal virus [23] (Figure 1A).

Ferrets belonging to the NS group presented mild histological lesion generally characterized by mild bronchopneumonia or bronchitis. On the contrary, the lung pathology observed in animals belonging to the S group showed signs of acute inflammation as diffuse alveolar damage, similar to the histological lesions presented on deceased human patients infected with the A(H1N1)pdm09 [24].

Recent studies performed in pigs showed interesting data on the immune responses, reflected on serum concentrations of acute phase protein as C-reactive protein, haptoglobin and serum amyloid A in response to IV [14,15]. In these studies, positive correlations were found between serum concentration of Hp and SAA and lung scores, and between clinical score and concentrations of SAA in IV infected pigs [14]. The results in this work are the first study in which Hp and SAA have been analysed in the context of IV in ferrets, providing further information of the acute phase proteins on the immune response to influenza infection. Our study suggested that there was an increase of Hp and SAA at day 2 pi (Figure 3A and B), however no statistical differences were found, probably due to the low number of animals per group. These results pave the way for analysing further APP responses to influenza infection in ferrets that could be useful for the ferret daily clinical practice.

In relationship to the antibody response, animals showed seroconversion from 10 dpi by ELISA and HIA in both infected groups. Interestingly, cross-reactive antibodies were generated, indicating that similar regions of each HA molecule was recognized by the immune system in ferrets. Noteworthy, HA sequence has only three changes at the aminoacid level when comparing M and F virus [9]. Interestingly, it has been reported that either single or double point mutations may alter epitopes of a particular antigenic site in the 2009 H1N1 pandemic virus HA [25].

Viral replication in the respiratory tract of the infected ferrets was directly associated to the more severe pathology observed. As expected, animals on S group showed higher viral titre (Figure 4) and higher tissue damage (Figure 2) which can be associated to the individual animal condition, but not to differential virulence in each strain, as it was the case in mice experiments [9].

Finally, it has been shown that detrimental innate cellular responses have been linked to up-regulation of several proinflammatory cytokines and chemokines and the down-regulation of IFNα in the lungs in ferrets infected with this two A(H1N1)pdm09 virus. Additionally, severe lung lesions were associated with greater up-regulations of pro-apoptotic markers and higher levels of apoptotic neutrophils and macrophages [26].

Our results suggested that the severity in the progress of infection was independent from the virus used for infection, which might be associated to the host immune response. Also, clinicopathological outcomes of A(H1N1)pdm09 infection in ferrets were not only due to viral replication abilities but also depended on the hosts’ capacities to mount efficient immune responses to control viral infection of the lung. Finally, ferret diversity in responding to A(H1N1)pdm09 may mimic the variability observed in the human population.
